# Development of diagnostic molecular markers for marker-assisted breeding against bacterial wilt in tomato

**DOI:** 10.1270/jsbbs.20027e

**Published:** 2022-06

**Authors:** Alebel Mekuriaw Abebe, Jinwoo Choi, Youngjun Kim, Chang-Sik Oh, Inhwa Yeam, Ill-Sup Nou, Je Min Lee

**Affiliations:** 1 Department of Horticultural Science, Kyungpook National University, Daegu 41566, South Korea; 2 Department of Horticultural Biotechnology, College of Life Science, Kyung Hee University, Yongin, Gyeonggi-do 17104, South Korea; 3 Department of Horticulture and Breeding, Andong National University, Andong, Gyeongbuk, 36729, South Korea; 4 Department of Horticulture, Sunchon National University, Suncheon, Jeonnam 57922, South Korea

Breeding Science 70: 462–473 (2020)

In the above article, there was a mistake in Table 3.

## False

The sequence of reverse primers was presented in the 3′→5′ direction (highlighted).

Table 3 False

## True

Now the sequences are reverse complemented and corrected in the 5′→3′ direction (highlighted).

Table 3 True

## Figures and Tables

**Table 3.** DNA marker information used in this study

Marker name	SNP position (bp)	Marker type		Primer sequence (5ʹ→3ʹ)	T_m_ (°C)	Restriction enzyme	Expected size (bp)	
							Susceptible	Resistant
RsR6-1	24,669,159	CAPS	F:	GGAAATATTGGTTACAATCCAGTG	57.5	*Mnl*I	227	173, 54
			R:	GAATACAACAAATCACTACCGGTC	59.3			
RsR6-2	34,389,374	CAPS	F:	CTTCTTGATAGGACGACGTGATAT	59.3	*Rsa*I	87, 116	203
			R:	CAATCAACGGATCACCCATTTTTC	59.3			
RsR6-3	34,399,541	CAPS	F:	CTCTTTTTGCCAGATCTTGAATAG	57.5	*Mnl*I	214	116, 98
			R:	CCATAGGTCAGCATCAAATTTCAA	57.5			
RsR6-4	35,950,028	CAPS	F:	GTTTTCCTTGCAAATCATTTTGGC	57.5	*Mse*I	116, 97	213
			R:	GTATATGTTGAGTTCACAATTCCC	57.5			
RsR6-5	37,049,726	CAPS	F:	CTCAGAAACTGGATAAACTCGAAG	59.3	*Hinf*I	204	129, 75
			R:	GGAGAAAGCAGCCAGCCATTTTT	60.6			
RsR6-6	37,186,202	dCAPS	F:	CGGTGATGAGCAGGATTGATAAAA	59.3	*Hpy*CH4III	234	200, 34
			R:	AGTCTTGGCCTTTGACGTGAAAGTGACACAAGAAG	60.6			
RsR12-1	2,941,301	dCAPS	F:	GTTACACGAACAAGCTTAAATTTCTAGATTTATCCC	58.8	*Aci*I	203	168, 35
			R	GTAATCAATTCGAAGGACCTGTC	64.9			

**Table 3.** DNA marker information used in this study

Marker name	SNP position (bp)	Marker type		Primer sequence (5ʹ→3ʹ)	T_m_ (°C)	Restriction enzyme	Expected size (bp)	
							Susceptible	Resistant
RsR6-1	24,669,159	CAPS	F:	GGAAATATTGGTTACAATCCAGTG	57.5	*Mnl*I	227	173, 54
			R:	GACCGGTAGTGATTTGTTGTATTC	59.3			
RsR6-2	34,389,374	CAPS	F:	CTTCTTGATAGGACGACGTGATAT	59.3	*Rsa*I	87, 116	203
			R:	GAAAAATGGGTGATCCGTTGATTG	59.3			
RsR6-3	34,399,541	CAPS	F:	CTCTTTTTGCCAGATCTTGAATAG	57.5	*Mnl*I	214	116, 98
			R:	TTGAAATTTGATGCTGACCTATGG	57.5			
RsR6-4	35,950,028	CAPS	F:	GTTTTCCTTGCAAATCATTTTGGC	57.5	*Mse*I	116, 97	213
			R:	GGGAATTGTGAACTCAACATATAC	57.5			
RsR6-5	37,049,726	CAPS	F:	CTCAGAAACTGGATAAACTCGAAG	59.3	*Hinf*I	204	129, 75
			R:	AAAAATGGCTGGCTGCTTTCTCC	60.6			
RsR6-6	37,186,202	dCAPS	F:	CGGTGATGAGCAGGATTGATAAAA	59.3	*Hpy*CH4III	234	200, 34
			R:	CTTCTTGTGTCACTTTCACGTCAAAGGCCAAGACT	60.6			
RsR12-1	2,941,301	dCAPS	F:	GTTACACGAACAAGCTTAAATTTCTAGATTTATCCC	58.8	*Aci*I	203	168, 35
			R	GACAGGTCCTTCGAATTGATTAC	64.9			

